# Hormones in experimental autoimmune encephalomyelitis (EAE) animal models

**DOI:** 10.1515/tnsci-2020-0169

**Published:** 2021-05-06

**Authors:** Majid Ghareghani, Amir Ghanbari, Ali Eid, Abdullah Shaito, Wael Mohamed, Stefania Mondello, Kazem Zibara

**Affiliations:** Neuroscience Laboratory, CHU de Québec Research Center and Department of Molecular Medicine, Faculty of Medicine, Laval University, Québec City, QC, Canada; Cellular and Molecular Research Center, Yasuj University of Medical Sciences, Yasuj, Iran; Biomedical and Pharmaceutical Research Unit and Department of Basic Medical Sciences, College of Medicine, QU Health, Qatar University, Doha, Qatar; Department of Biological and Chemical Sciences, Faculty of Arts and Sciences, Lebanese International University, Beirut, Lebanon; Clinical Pharmacology Department, Menoufia Medical School, Menoufia University, Shibin Al Kawm, Egypt; Department of Basic Medical Sciences, Kulliyyah of Medicine, International Islamic University Malaysia (IIUM), Kuantan, Pahang, Malaysia; Department of Biomedical and Dental Sciences and Morphofunctional Imaging, University of Messina, Messina, Italy; PRASE, Lebanese University, Beirut, Lebanon; Biology Department, Faculty of Sciences – I, Lebanese University, Beirut, Lebanon

**Keywords:** multiple sclerosis, experimental autoimmune encephalomyelitis, EAE, endocrine system, hormone

## Abstract

Multiple sclerosis (MS) is an inflammatory demyelinating disease of the central nervous system (CNS) in which activated immune cells attack the CNS and cause inflammation and demyelination. While the etiology of MS is still largely unknown, the interaction between hormones and the immune system plays a role in disease progression, but the mechanisms by which this occurs are incompletely understood. Several *in vitro* and *in vivo* experimental, but also clinical studies, have addressed the possible role of the endocrine system in susceptibility and severity of autoimmune diseases. Although there are several demyelinating models, experimental autoimmune encephalomyelitis (EAE) is the oldest and most commonly used model for MS in laboratory animals which enables researchers to translate their findings from EAE into human. Evidences imply that there is great heterogeneity in the susceptibility to the induction, the method of induction, and the response to various immunological or pharmacological interventions, which led to conflicting results on the role of specific hormones in the EAE model. In this review, we address the role of endocrine system in EAE model to provide a comprehensive view and a better understanding of the interactions between the endocrine and the immune systems in various models of EAE, to open up a ground for further detailed studies in this field by considering and comparing the results and models used in previous studies.

## 1 Introduction

The endocrine system (ES) consists of numerous glands which regulate many physiological processes by producing hormones throughout the body [1]. The interconnections between the ES and the central nervous system (CNS) are significant since both are involved in maintaining homeostasis whereby fluctuations in the ES could cause pathological situations in the CNS [2]. Over the past 40 years, it has been reported that activation of the immune system is associated with alterations in the neuroendocrine functions, indicating that its functions are under the supervision of the CNS [3]. ES hormones can bind to the receptors on a wide range of cell types ranging from immune cells to neural cells which would affect the function of these cells.

The therapeutic activity of hormones has been investigated in multiple sclerosis (MS), an inflammatory demyelinating disease of the CNS in which activated immune cells attack the CNS. Although the symptoms are different from patient to patient, all patients have demyelination in common. Demyelination interferes with normal signal conduction along neuronal axons, ultimately resulting in a number of clinical symptoms affecting the autoreactive immune responses and myelination [4]. Depending on the manner by which the disease attacks the body over time, reversible/irreversible sequelae would develop. (A) Relapsing-remitting MS (RRMS) is the most common kind of MS in which the severity of the disease is followed by partial or complete recovery, known as remission. This remission is itself followed by relapses and exacerbations of symptoms, a cycle that is repeated over time. (B) Secondary-Progressive MS (SPMS); patients do not show the relapse/remission cycle; instead, the disease severity progresses steadily. (C) Primary-Progressive MS (PPMS) comprises a small group of MS patients in which a steady accumulation of disabilities occurs without attacks. (D) Progressive-Relapsing MS (PRMS) is another rare kind in which the disease severity worsens steadily, with acute relapses but without remissions [5]. Although the etiology of MS is still not known, a wide range of factors have been implicated in the pathogenesis of the disease including genetic risk factors, environmental risk factors such as Epstein-Barr Virus, sunlight, vitamin D, and smoking, and lifestyle risk factors such as diet and microbiota. This has been nicely reviewed by Nourbakhsh and Mowry [6].

Animal models such as Experimental Autoimmune Encephalomyelitis (EAE) have been extensively used to investigate the potential therapeutics for MS [7]. While the sensitization to autoantigens naturally happens due to an unknown mechanism, the EAE model needs an external immunization [8]. EAE is an organ-specific cell-mediated autoimmune demyelinating disease of the CNS in which macrophages and T lymphocytes mediate the injury to myelin sheath. Considering the multifactorial nature of MS etiology and the complexity of the inflammatory and immunological processes, several protocols have been used for EAE induction in mice and rats [9,10]. There are some recognized antigens in MS, which act as a target of B and T cell response, such as myelin oligodendrocyte glycoprotein (MOG), myelin basic protein (MBP), myelin-associated glycoprotein (MAG), and proteolipid protein (PLP) [11]. These peptide antigens are emulsified in Complete Freund’s Adjuvant (CFA), an enhancer of autoantigens’ immunogenicity in EAE induction, and then subsequently injected to induce demyelination and neuroinflammation in mice. In addition, this model needs another agent, Pertussis toxin (PT), to open up the blood-brain barrier and facilitates the migration of pathogenic T-cells into the CNS [12].

This review covers endogenous hormonal alterations involved in immune cell functions and demyelination in EAE. Pharmacologically, this review includes exogenous hormonal administration that can influence EAE. This review brings many hypotheses and ideas to the attention of the reader to fill the current gaps in the existing studies to ultimately reach a thorough understanding of the role of all hormones in EAE onset and development. It is worth noting that some hormones have conflicting effects in the EAE model. It is not clear whether this is due to age, gender, immunogen, or strain of the EAE animal model. We categorized in separate tables all the studies mentioned in this review according to the above-mentioned factors, indicating at the end of each endocrine system the hormones that have been studied in the EAE animal models.

## 2 The pituitary gland

### 2.1 Anterior lobe of pituitary

#### 2.1.1 Luteinizing hormone (LH)

Increased serum level of LH has been demonstrated in male, but not female, passive EAE mice, compared to control mice [13]. This suggested that gonadal dysfunction occurs in males during passive EAE, but not females. Importantly, ovariectomized mice showed significantly higher level of LH in this group.

#### 2.1.2 Prolactin (PRL)

Riskind et al. investigated prolactin in response to EAE induction and bromocriptine, a dopamine agonist. Elevated levels of prolactin were found after immunization with EAE induction emulsion and before EAE onset [14]. Importantly, bromocriptine not only suppressed the severity of EAE, but also inhibited the secretion of prolactin both in serum and pituitary. This suggested that prolactin is involved in immune response during EAE. Moreover, plasma prolactin was markedly suppressed by bromocriptine treatment initiated after the onset of EAE symptoms [15]. To support this idea, Canonico et al. treated Lewis rats, 2 days ahead of EAE induction, with dihydroergocryptine, a natural alkaloid derivative which exhibits D2 dopaminomimetic properties [16]. They demonstrated that dihydroergocryptine medication markedly reduced the prolactin level, compared to untreated-EAE rats, which also led to amelioration of EAE severity.

Since dopaminergic agonists therapy in EAE reduces the severity of disease and prolactin level, Esquifino et al. investigated whether ectopic pituitary would affect EAE and prolactin [17]. Therefore, pituitary grafting from littermate donors was performed and then rats were immunized by EAE induction emulsion. Only sham-operated rats exhibited EAE symptoms, whereas prolactin plasma level started to increase from the first day postimmunization. In contrast, prolactin decreased in pituitary-grafted rats and reached its lowest level on day 15 after immunization, without showing any signs of EAE symptoms. In another group of experiments, bromocriptine-treated rats did not show any sign of EAE and also had very low concentration of prolactin in the plasma, whereas prolactin was constantly high in untreated-EAE rats, which showed first sign of EAE symptoms on day 15. In summary, low level of circulating prolactin coincided with absence of clinical symptoms of EAE. However, Costanza et al. challenged this common view and reported, using PRL-deficient (Prl^−/−^) and PRL receptor-deficient (Prlr^−/−^) mice, that prolactin is not required for the development of EAE [18]. Indeed, PRL or PRLR couldn’t impair the development of Th1 and Th17 responses against MOG35-55, but only delayed their generation before a full clinical severity was observed, similar to wild-type EAE mice. Interestingly, it has been recently claimed that prolactin synergistically enhances the efficiency of interferon-β in amelioration of EAE severity, while prolactin alone did not affect EAE development. These findings introduced prolactin as a beneficial factor to increase the therapeutic potential of interferon-β in MS [19]. In contrast, another study suggested that inhibition of elevated prolactin by antigen-presenting cells ameliorates the EAE severity. Taking into account that induction of pathogenic eomesodermin-positive CD4^+^ T cells is associated with a transition from an acute stage to a later stage in EAE, Zhang et al. showed that the expression of prolactin is enhanced in late EAE lesions by B cells and MHC class II^+^ myeloid cells [20].

#### 2.1.3 Growth hormone (GH)

EAE can be significantly modified by caloric restriction since calorie restricted EAE rats showed significantly reduced levels of GH, compared to controls [21]. To distinguish the role of GH and GH-releasing hormone (GHRH) in EAE development, Shohreh et al. used a GHRH knockout mouse (GHRH^−/−^) to induce EAE [22]. Results showed that untreated and GHRH super agonist JI-38-treated GHRH^−/−^ mice had lower susceptibility to EAE, compared to control and GH-treated GHRH^−/−^ mice. Importantly, GH-treated GHRH^−/−^ mice showed severe clinical scores, compared to controls, associated with a considerable increase in spleen size and T-cell proliferation specific to MOG peptide. Therefore, GH plays a key role in the development of EAE, but not GHRH.

#### 2.1.4 Adrenocorticotropic hormone (ACTH)

The neurotrophic peptide [H-Met(O_2_)-Glu-His-Phe-D-Lys-Phe-OH], a synthetic analog of ACTH [4,5,6,7,8,9], was used by Duckers et al., to study its effect in EAE model. They found that repeated subcutaneous injections of this analog suppressed the development of EAE-related clinical symptoms, improved motor performance, and reduced the reaction time upon thermal stimulation, in comparison to untreated-EAE mice [23]. This analog also protected against the development of EAE symptoms by improving the visual evoked potentials of animals suffering from EAE [24].

Weidenfeld et al. examined the effect of linomide (quinoline-3-carboxamide), a novel synthetic compound with various immunomodulatory properties, on adrenalectomized EAE rats. Linomide-treated EAE-adrenalectomized rats did not develop any signs of EAE, in comparison to untreated EAE-adrenalectomized rats which showed high EAE clinical scores. Moreover, a marked reduction in thymus weight was seen in the linomide-treated, compared to untreated, EAE-adrenalectomized rats [25].

The hormonal change in EAE was also investigated in Lewis (LEW), Brown Norway (BN), and Dark Agouti (DA) rat strains. Basal ACTH levels were found in BN and DA rats, whereas lower levels were observed in LEW rats. These strains showed different susceptibilities to EAE induction [26]. On the other hand, although some studies reported a slight insignificant increase in ACTH plasma level in EAE Lewis rat, compared to control [27,28,29,30], Ruocco et al. showed a sharp and significant increase in ACTH in the same animal model [31].

Treatment with Acthar gel, an FDA-approved medication for acute-MS and a highly purified gel preparation of porcine ACTH1-39 [32], was used in female EAE mice. This gel ameliorated the severity of EAE clinical scores and protected EAE by reducing immune cell infiltration and demyelination of spinal cord. In addition, activated microglia and macrophages were measured via Ricinus communis agglutinin (RCA)-I lectin histochemistry and found fewer RCA-1^+^ cells in the spinal cords of Acthar-treated mice compared to untreated-EAE mice at, or after, the onset of relapse. Moreover, Acthar gel treatment markedly suppressed *ex vivo* myelin peptide-induced CD4^+^ T cell proliferation [33], as activation of T cells reactive to MBP was found in MS patients compared to healthy subjects [34]. In accordance, Brod and Hood also reported a two-fold increase in T regulator cells (Tregs) in the periphery of MOG-induced C57BL/6 EAE ACTH-fed mice, in comparison to control; hence, ACTH is able to shift the Th1/Th17 CD4^+^ T cells to the Th2/Treg phenotype [35]. Recently, it has been shown that oral ACTH therapy in EAE caused a reduction in the expression of CD11b^+^, IL-6, and IL-17 producing lymphocytes in the lamina propria (LP), where the majority of cells involved in immune reactions are located. Indeed, they observed a reduction in IL-17 and IFN-γ, while CD4^+^ FoxP3^+^ Treg cell frequency increased [36].

#### 2.1.5 Thyroid-stimulating hormone (TSH) and follicle-stimulating hormone (FSH)

No studies were found concerning TSH and FSH in EAE model.

### 2.2 Posterior lobe of pituitary

#### 2.2.1 Vasopressin

Induction of EAE does not significantly alter the plasma level of vasopressin. In fact, a single injection with IL-1b in EAE Lewis rats, 1 week prior to immunization with EAE induction emulsion, decreased the severity of EAE; however, it also significantly increased the plasma vasopressin, compared to untreated-EAE rats [27]. In accordance, Ruocco et al. reported an elevated content of the hypothalamic arginine vasopressin in male Lewis EAE rats, compared to controls [31]. Moreover, Quintanar-Stephano et al. showed that administration of desmopressin, an agonist of arginine vasopressin, markedly ameliorates the severity of EAE rats as measured by decreased clinical scores and immune cell infiltration into the spinal cord, suggesting that arginine vasopressin could have an important role in the maintenance of immune competence [29]. A recent study on Lewis rats showed that low levels of vasopressin or inhibition of its receptor by conivaptan, an antagonist, resulted in the reduction in disease severity. This was associated with a decrease in BBB permeability through V1a and V2 vasopressin receptors [37].

#### 2.2.2 Oxytocin

Recently, a study on EAE rats investigated the changes in expression levels of hypothalamic feeding-related peptide genes and neuroendocrine responses and showed an activation in the oxytocinergic pathway as they observed an increase in mRNA and plasma oxytocin in EAE rats [38].

A summary of EAE animal models used to study hormones in the pituitary gland is given in Table 1.

**Table 1:** A summary of EAE animal models used for hormones in the pituitary gland

Tissue	Source	Hormone	Animal and strain of EAE model	Gender	Age	Immunogen for EAE induction	Reference
The Pituitary Gland	Anterior lobe	Luteinizing hormone (LH)	SJL/J mice	M & F	8–12 weeks	PLP139-151	[13]
		Prolactin (PRL)	LWS rat	F	60 days	gpSCH	[14]
			LWS rat	M	NA	gpSCH	[15]
			LWS rat	M	6 weeks	wrSCH	[17]
			GMM	F	8–12 weeks	MOG35-55	[18]
			Virgin C57BL/6 mice	F	8–10 weeks	MOG35-55	[19]
			C57BL/6 mice	F	6–8 weeks	MOG35-55	[20]
		Growth hormone (GH)	LWS rat	M	6 weeks	wrSCH	[21]
			GMM	F	6 weeks	MOG35-55	[22]
		Adrenocorticotropic hormone (ACTH)	LWS rat	F	NA	gpSCH	[23]
			LWS rat	M	NA	gpSCH	[24]
			Sabra rats	M	NA	wrSCH	[25]
			LWS, BN, and DA rats	F	8–12 weeks	gpMBP & rrMOG	[26]
			LWS rat	M	NA	gpMBP	[27]
			LWS rat	M	NA	gpBH	[28]
			LWS rat	F	NA	gpBH	[29]
			C57BL/6 mice	F	6–9 weeks	MOG35-55	[30]
			LWS rat	M	6–8 weeks	gpMBP	[31]
			SJL/J mice	F	4–6 weeks	PLP139-151	[33]
			C57BL/6 mice	F	6–8 weeks	MOG35-55	[34]
			C57BL/6 mice	F	6–8 weeks	MOG35-55	[36]
	Posterior lobe of pituitary	Vasopressin	LWS rat	M	NA	gpMBP	[27]
			LWS rat	F	NA	gpBH	[29]
			LWS rat	M	6–8 weeks	gpMBP	[31]
			LWS rat	F	10–12 weeks	gpBH	[37]
		oxytocin	LWS rat	M	7 weeks	gpMBP	[38]

Abbreviations: gpSCH – guinea pig Spinal Cord Homogenate; gpMBP – guinea pig Myelin Basic Protein; gpBH – guinea pig Brain Homogenate; wr-SCH – wistar rat Spinal Cord Homogenate; rrMOG – recombinant rat Myelin Oligodendrocyte Glycoprotein; PLP – Proteolipid Protein; DA – Dark Agouti rats; BN – Brown Norway; SJL/J – Swiss Jackson Laboratory/Jackson mice; GMM – Genetically manipulated mice.

## 3 Hypothalamus

### 3.1 Thyrotropin-releasing hormone (TRH)

Early investigation showed that injury in TRH-containing axons occurs early in the development of EAE rats [39]. Later, Brod and Bauer examined whether TRH has therapeutic activity in MS using active and adaptive EAE mouse model [40]. Results revealed that oral administration of TRH markedly ameliorates the severity of EAE scores and symptoms by reducing Th1-like, Th17, and TNF-α cytokines and increasing Th2-like cytokines of IL-13 in spinal cord lymphocytes. Therefore, TRH was introduced as a hormone with therapeutic potential in MS.

### 3.2 Gonadotropin-releasing hormone (GnRH)

Quintanar et al. investigated the role of GnRH in EAE model since GnRH has neurotrophic properties and the spinal cord possesses GnRH receptor [41]. GnRH therapy considerably reduced the clinical scores of EAE and increased the number of larger area axons in GnRH-EAE rats, compared to untreated-EAE rats. In addition, GnRH-treated EAE rats showed increased levels of expression of neurofilaments NF-68, NF-160, and NF-200 compared to untreated-EAE rats, whereas the latter showed higher NF-68 and NF-160 levels compared to control rats. On the other hand, mice treated with GnRH and Antide, a GnRH receptor antagonist, showed similar areas to the rats without GnRH therapy. Moreover, GnRH^+^ Antide-EAE mice showed similar levels of NFs to untreated-EAE group. Furthermore, Guzman-Soto et al. investigated the role of leuprolide acetate, a GnRH agonist, in EAE model of MS. Results confirmed that leuprolide acetate reduced the severity of EAE scores while significantly increasing the expression of NF-68 and NF-160, compared to untreated-EAE mice [42]. This was coincident with higher levels of MBP protein expression and fewer numbers of infiltrated cells in the spinal cord. Leuprolide acetate seemed to be more efficient than GnRH in inducing the expression of NFs. Later, leuprolide acetate-treated EAE rats were demonstrated to have a decline in the activation of NF-κB, a transcription factor mainly involved in inflammation, immune response, and cell survival, but also proinflammatory mediators including TNF-α, IL-1β, and IL-17A, in comparison to untreated-EAE rats [43].

### 3.3 Growth hormone-releasing hormone (GHRH)

The potential involvement of GHRH in the pathogenesis of EAE was addressed using GHRH receptor-deficient mice (Ghrhr^lit/lit^) which showed no clinical symptoms of EAE during the entire period of study [44]. In addition, GHRHR deficiency did not affect the activation of T cells and GHRHR-deficient EAE mice had higher levels of anti-MOG IgG, compared to control, suggesting that resistance of GHRHR^−/−^ mice to EAE could be due to a deficient immune response. Therefore, GHRH plays an important role in the development of EAE and could be a therapeutic target.

### 3.4 Corticotropin-releasing hormone (CRH)

The hypothalamo-pituitary-adrenal axis (HPA-axis) activity is regulated by CRH and arginine vasopressin (AVP) in the paraventricular nucleus (PVN) of the hypothalamus [45]. Calzà et al. showed a reduction in CRH mRNA levels in the PVN 4 days post EAE induction, which recovered at 10 days but decreased again until day 20, the last day of experiment [46]. In contrast, Huitinga et al. showed unchanged CRH levels in the median eminence of male EAE Lewis rats, compared to controls [27]. On the other hand, CRH-deficient mice were found to be resistant to EAE induction and developed a mild form of EAE [47]. Moreover, administration of Astressin, a specific CRH antagonist, reduced the disease severity in mice. Indeed, CRH’s effects on EAE development were shown to be independent of its glucocorticoid-inducing effect. In addition, results showed that IκBα phosphorylation, which inhibits the NF-κB transcription factor, was decreased in CRH-deficient T cells. Finally, peripheral CRH was suggested to apply its proinflammatory activity in EAE through a selective increase in Th1-type responses.

### 3.5 Somatostatin (SST)

Muhvic and his colleagues introduced the role of somatostatin in EAE since 1992 [48]. On the other hand, they investigated the effect of the somatostatin analogue SMS 201-995 (octreotide) on EAE in the relatively resistant Albino Oxford (AO) strain of rats [49]. Results showed that the number of EAE-induced rats was higher in somatostatin-treated rats compared to controls. Moreover, the proportion of CD8^+^ T cells in the popliteal lymph nodes (PLN) decreased in somatostatin-treated rats, in comparison to control, while the proportion of CD25^+^ cells increased. Therefore, somatostatin increased the susceptibility of AO rats to EAE. In contrast, another study in mice, instead of AO rats, showed that oral somatostatin inhibits EAE [50]. Indeed, the authors showed a reduction of Th1 and Th17 and an induction of Th2-like IL-4 cytokines in the spleen and CNS which caused an inhibition of the disease. Further investigations are needed to clarify the role of somatostatin in EAE.

A summary of EAE animal models used to study hypothalamus hormones is given in Table 2.

**Table 2:** A summary of EAE animal models used for hormones in the hypothalamus and pineal gland

Tissue	Hormone	Animal and strain of EAE model	Gender	Age	Immunogen for EAE induction	Reference
Hypothalamus	Thyrotropin-releasing hormone (TRH)	LWS rat	M	NA	lrSCH	[39]
		C57BL/6	F	6–8 weeks	MOG35-55	[40]
	Gonadotropin-releasing hormone (GnRH)	LWS rat	F	NA	gpBH	[41]
		LWS rat	F	3–4 months	gpBH	[42,43]
	Growth hormone-releasing hormone (GHRH)	GMM	F	6 weeks	MOG35-55	[44]
	Corticotropin-releasing hormone (CRH)	LWS rat	F	NA	gpSCH	[46]
		LWS rat	M	NA	gpMBP	[27]
		GMM	NA	6–8 weeks	MOG35-55	[47]
	Somatostatin (SST)	DA & OA rats	M	Adult	CFWMH	[48]
		AO rats	M	2–3 months	bBH	[49]
		C57BL/6 mice	F	6–8 weeks	MOG35-55	[50]
Pineal gland	Melatonin	(SJL × PL/J) F1 mice	NA	12 weeks	gpSCH	[51]
		LWS rat	M & F	8–12 weeks	rSCH	[52]
		C57BL/6 mice	F	8 weeks	MOG	[53]
		C57BL/6 mice	F	6–8 weeks	MOG35-55	[54]
		LWS rat	F	5–6 weeks	GPSCH	[55]
		C57BL/6 mice	F	6–8 weeks	MOG35-55	[56]
		C57BL/6 mice	F	6–8 weeks	MOG35-55	[57]

Abbreviations: gpSCH – guinea pig Spinal Cord Homogenate; gpBH – guinea pig Brain Homogenate; lrSCH – lewis rat Spinal Cord Homogenate; CFWMH – Calf Brain White Matter Homogenate; bBH – bovine Brain Homogenate; rSCH – rat Spinal Cord Homogenate; DA – Dark Agouti rats; AO – Albino Oxford; GMM – Genetically manipulated mice.

## 4 Pineal gland

### 4.1 Melatonin

Melatonin was investigated in EAE for the first time by Constantinescu et al., when the effect of luzindole, a melatonin receptor antagonist, was examined [51]. In fact, luzindole inhibited the development of EAE through inhibition of melatonin. Later, several studies showed that EAE severity is reduced by melatonin therapy [52,53,54]. The reduced level of EAE scores in melatonin-treated group was coincident with lower number of inflammatory cells infiltration (CD4, CD8, NK) into the spinal cord [52]. They also found that only ICAM-1 expression, but not LFA-1a, is considerably decreased by melatonin therapy. Since NK cells play a suppressive role in EAE, it’s suggested that melatonin reduces the severity of EAE through a mechanism involving ICAM-1.

On the other hand, the effects of melatonin therapy on T effector/regulatory responses of EAE showed an increased level of Treg frequency and IL-10 synthesis in the CNS, whereas Th1/Th17 responses in both peripheral and CNS were reduced in melatonin-treated EAE mice [53]. Moreover, melatonin reduced the frequencies of T effector and T central memory cells, and TNF-producing CD4^+^ cells. In addition, mononuclear infiltration (CD4 and CD11b cells) in the CNS was also reduced by melatonin therapy. This suggested that melatonin ameliorates EAE by controlling both peripheral and central T effector/regulatory responses. Further work by Chen et al. showed, however, that the percentage of sorted splenic dendritic cells and the population of Foxp3^+^ CD4^+^ T cells in the spleens were not significantly different between groups [54]. Furthermore, melatonin enhanced splenic IL-10 in regulatory T cells by inducing IL-27 expression in the splenic dendritic cells which suppresses the expression level of IFN-γ, IL-17, IL-6, and CCL20 in the CNS and inhibits antigen-specific T cell proliferation. In line, Ghareghani et al. reported for the first time that the age of EAE models plays a substantial role in melatonin therapy [55]. Indeed, as the administration of melatonin in EAE Lewis rats exacerbated the clinical symptoms of EAE by enhancing the immune cell infiltration into the CNS, demyelinated plaques (low MBP-positive cells) activated astrocytes, serum lactate, and the ratio of IFN-γ/IL-4. Our study on EAE mice showed that melatonin alone or in combination with baclofen reduces clinical scores and demyelination which is associated with an increase in IL-4 and a decrease in IFN-γ serum levels. Furthermore, catalase and superoxide dismutase, as antioxidant enzymes, increased following the treatment, while the oxidative stress-related malondialdehyde levels decreased [56]. We further showed that melatonin therapy modulates cerebral metabolism and enhances remyelination which is associated with an increase in pyruvate dehydrogenase kinase-4 (PDK-4), an enzyme involved in fatty acid synthesis during remyelination, which we believe is a side effect of melatonin therapy [57]. Further studies are needed to determine the precise role of melatonin in the EAE model, especially with respect to age, gender, strain of mice/rats used, and melatonin dosage.

A summary of EAE animal models used to study pineal gland hormones is given in Table 2.

## 5 Thyroid gland

### 5.1 Thyroxine (T4)

The effects of thyroid hormones are predominantly mediated by thyroid hormone receptors (THRs), which are part of the family of nuclear receptors. The 2 receptor genes THRA and THRB encode the 2 proteins THRα and THRβ, respectively. In addition, each of these genes generates two isoforms (α1-2, β1-2) by alternative spicing. The α isoforms are widely expressed in the fetal brain as well as oligodendrocyte precursor cells (OPCs), while the β isoforms expression is more restricted to differentiated oligodendrocytes with a dramatic increase that starts at birth in the rat [58,59].

Investigating the effect of thyroid hormone (thyroxine, T4) on EAE mice showed that EAE induction caused a sharp increase in the number of Ki-67^+^ and BrdU^+^ nuclei, a marker of proliferating cells, in the SVZ, white and gray matter of brain and spinal cord, and between ependymal cells in the central canal. Few numbers of Ki-67^+^ and BrdU^+^ cells belong to perivascular inflammatory cells and parenchymal glial cells, which are strongly expressed in the acute stage of EAE. In contrast, T4 administration reduced the rate of proliferation and undifferentiated precursors in the spinal cord and SVZ. Moreover, codistribution of Ki-67 and nestin immunoreactivity showed a reduction in the expression of nestin-positive cells in the SVZ. Moreover, they showed that the administration of thyroid hormone in EAE reduces the rate of proliferation, while it favors differentiation towards oligodendrocytes and their maturation; hence, T4 therapy ameliorates the regulation of myelin-forming proteins in EAE model. Importantly, the increased level of MBP in EAE model was found to be caused not only by a direct regulation of MBP gene expression, but also by newly formed oligodendrocytes [60]. The fact that MBP is a target gene for thyroid hormone receptor has been already reported by Farsetti et al. [61].

In line, Gallo and Armstrong reported that the level of nestin protein expression is high in proliferating progenitors, while it declines in differentiated oligodendrocytes [62]. In support of these findings, Lezoualc’h et al. demonstrated that the nestin promoter has a binding site for nuclear receptors, which allows the thyroid hormone to bind nestin in neural progenitor cells of the embryonic CNS [63].

Albornoz et al. induced EAE in the gestated mice under hypothyroidism which already were treated with or without T4. Their findings showed higher clinical score of EAE, demyelination area, immune cell infiltration, the number of CD4^+^ and CD8^+^ T cells, percentage of death in oligodendrocytes (labeled with TUNEL) in the spinal cord, and lower expression of myelin basic protein (MBP) of EAE mice gestated in hypothyroid mothers, compared to EAE mice gestated in normal mother. All of these changes reverted when T4 was added during gestation [64]. This suggested that gestational hypothyroidism causes more severe inflammation and demyelination in EAE mice. Given the fact that Nerve Growth Factor [30] drops in acute phase of EAE [46], Calza et al. reported that T4 therapy restored the NGF level. NGF administration showed a beneficial role by diminishing the clinical score of EAE and by protecting oligodendrocytes against injury induced by TNF-α proinflammatory cytokine [65]. Moreover, several studies investigated the role of nuclear hormone receptor superfamily members that are known to affect the immune response, mainly the receptors for retinoids, steroids, and thyroid hormone. Results revealed an expression of the peroxisome proliferator-activated receptors (PPARs) in the inflammatory infiltrate of EAE mice spinal cord. In contrast, administration of PPAR-γ ligand 15d-PGJ2 reduced the severity of EAE by inhibiting the activation and expansion of encephalitogenic T cells, which suggests that PPAR-γ agonists may act as a new therapeutic strategy for MS in which thyroid hormones are most probably involved [66,67,68]. Furthermore, Fernandez et al. showed that T4 treatment is effective in reducing the severity of the relapse in Lewis, but not Dark Agouti (DA), rats of EAE model. They also showed that T4 favors myelin sheath reconstruction by increasing the mRNA expression levels of platelet-derived growth factor α receptor (PDGFαR) marker of the oligodendrocyte precursor cells (OPC) in the spinal cord of EAE-DA rats on day 18 postimmunization [69]. More details about the role of TH on oligodendrocytes have been reviewed by Calzà et al. [70].

Two types of circulating TH are present: L-triiodothyronine (T3), the active form, and tetra-iodothyronine (T4), the precursor of T3. Deiodinase enzymes (D) type 1 and 2 catalyze conversion of T4 to T3, whereas D type 3 (D3) catalyzes inactivation of T4 and T3 into reverse (r)T3 and 3,3-diiodothyronine (T2), respectively [71,72]. Since D3 expression may play an important role in decreasing the local bioavailability of TH at the inflammation sites, Boelen et al. showed that D3 is considerably increased in the inflammatory sites of EAE mice spinal cords [71]. While previous studies reported that most of the cellular effects of T3 are mediated by THRα and THRβ [73,74], Fernández et al. showed that THRα-1 and THRβ-1 mRNA expression are markedly increased in proliferating neurospheres media isolated from SVZ of adult EAE rats; however, THRα-2 expression did not show any significant changes. Moreover, treatment by T3 increased the expression of oligodendrocyte transcription factor Olig-1, a basic helix–loop–helix (bHLH) transcription factor expressed in cells of the oligodendrocyte lineage, and stimulated oligodendrocytes maturation. Indeed, T3 increases more than 3- and 10- fold the generation of GFAP^+^ astrocytes and CNPase^+^ oligodendrocytes when used during proliferation and differentiation conditions [75]. Importantly, this elevated expression of astrocytes favors the conversion of T4 to T3 by D2 and benefits an increased bioavailability of the active T3 and could also be detrimental for myelin repair [72,75].

On the other hand, the effects of T3 on EAE were investigated by D’Intino et al., using the nonhuman primate Callithrix Jacchus (marmoset), instead of EAE mice. Clinical scores of EAE severity were significantly decreased by T3 therapy; however, there was a significant increase in the mRNA expression levels of PDGFαR in the spinal cord and MBP in the optic nerve and cerebral cortex of T3-treated EAE group. Moreover, D3, but not D2, mRNA levels were significantly increased by T3 therapy, compared to untreated EAE. While THRβ-1 and THRβ-2 significantly elevated in T3-EAE, THRα-1 and THRα-1 did not show significant changes in response to T3 therapy, compared to EAE [76]. A year later, Dell’Acqua et al. showed that T3 therapy causes a better outcome of clinical and neurophysiological parameters; however, the plasma levels of T3 and T4 did not show significant differences between EAE and EAE-T3 groups on day 39 postimmunization. Indeed, T3 therapy did not affect inflammatory cellular infiltrate, but it strongly suppressed the loss of myelin staining, increased myelination, and did not present any nude axons, compared to untreated EAE. Then, they also demonstrated that the dysregulation of THR expression in the EAE was corrected by T3, as THRα-1 and THRβ expression significantly decreased in T3-treated mice, although THRα-2 showed no significant differences [77]. Finally, Castelo-Branco et al. showed that treatment of MBP63–88-specific T cell lines with T3, prior to transfer to naïve rats to establish passive EAE model, had no effect on T cell proliferation, but significantly decreased the frequency of IL-17-producing cells and increased the expression of C–C chemokine receptor type 7 (Ccr7) and L-selectin (D62L) [78]. CCR7 ligands have a novel function in stimulating the pathogenic Th17 cells in EAE induction through IL-23-depedent generation from dendritic cells [79], while CD62L regulates naïve T cell homing into lymph nodes and the migration of leucocytes to sites of inflammation [80]. Castelo-Branco et al. suggested that T3 has the potential to inhibit lymphocytes migration from lymph nodes into the CNS [78]. Investigation of the adult offsprings gestated in hypothyroxinemia demonstrated that it leads to imprint the immune response and subsequently early appearance of EAE symptoms [81].

### 5.2 Calcitonin (CT)

CT therapy was shown to delay the onset of EAE, while its combination administration with 1,25(OH)_2_D3 lowered the effective dose required to suppress EAE. Importantly, this combination therapy is dependent on dietary calcium [82]. However, at the molecular level, CT administration does not seem to be a necessary factor for 1,25(OH)_2_D3-mediated suppression of EAE. Indeed, knock-out mice for CT and calcitonin gene-related peptide-α (CGRP) genes did not change the EAE progression or serum calcium, while administration of 1,25(OH)_2_D3 significantly ameliorated the EAE severity and enhanced the serum level of calcium [83].

Moreover, it has been reported that SA13353, a novel transient receptor potential vanilloid 1 (TRPV1) agonist, and capsaicin (Cap) elevate the serum level of CGRP [84]. The therapeutic activity of CGRP was then examined by Matsuda et al., using a human CGRP-expressing plasmid transfected to C57BL/6 mouse bone marrow-derived matured DC (mDC) followed by the induction of EAE in this mouse. It was observed that only 50% of mice developed EAE in the *CGRP*-transfected group, compared with 80% in the mock-transfected group. In addition, cytokines production of IL-10, IL-2, IL-6, IL-17, TNF-α, and IFN-γ from spleen cells of EAE mice increased in CGRP-transfected mice, which was dependent on MOG concentration, compared with the mock-transfected mice. Moreover, the proportion of CD4^+^ CD25^+^ Foxp3^+^ cells increased in the *CGRP*-transfected mice, suggesting that gene therapy with CGRP-expressing mDC would be a novel strategy to suppress the development of EAE [85].

Since CGRP binds to its specific receptor composed of calcitonin receptor-like receptor (CLR) and receptor activity-modifying protein 1 (RAMP1) [86], Mikami et al. examined the role of CGRP in EAE using RAMP1-deficient mice. Although a lower EAE score and delayed peak score were observed in RAMP1^−/−^ mice, compared with wild-type, the population of Th17 cells was also decreased in the first group suggesting that CGRP promotes Th17-mediated inflammation by increasing IL-17 and IL-21 production in EAE mice [87]. Finally, CGRP-deficient mice showed aggravated EAE signs, while injection of CGRP into the lumbar CSF decreased EAE severity. Moreover, the production of TNF-α, IFN-γ, IL-17, IL-2, and IL-4 did not demonstrate any alterations in the peripheral lymphocytes of CGRP-treated mice; however, it reduced microglia transition to bushy morphology. Indeed, the expression of Iba1^+^ microglia showed slightly more bipolar and less round-fitting morphology in CGRP-treated mice which suggests that CGRP can inhibit microglia activation in EAE [88].

A summary of EAE animal models used to study thyroid hormones is given in Table 3.

**Table 3:** A summary of EAE animal models used for hormones in the thyroid and parathyroid glands

Tissue	Hormone	Animal and strain of EAE model	Gender	Age	Immunogen for EAE induction	Reference
Thyroid Gland	Thyroxine (T4)	C57BL/6 mice	F	6–9 weeks	MOG35-55	[30]
		LWS rat	F	NA	gpSCH	[46]
		LWS rat	F	NA	gpSCH	[60]
		C57BL/6 mice	F	6–8 weeks	MOG35-55	[64]
		Callithrix Jacchus (marmoset)	NA	NA	rrMOG	[65]
		GMM	NA	7–10 weeks	MBP Ac1-11	[66]
		GMM	NA	NA	MOG35-55	[68]
		LWS & DA rats	F	NA	gpSCH	[69]
		DA rat	F	7–8 weeks	rSCH	[71]
		LWS rat	F	2–3 months	gpSCH	[75]
		Callithrix Jacchus (marmoset)	M & F	11–144 months	hrMOG1-125	[76]
		DA rats	F	NA	gpSCH	[77]
		DA/Kini rats	M & F	NA	rMOG1-125 & gpMBP63-88	[78]
		GMM	NA	NA	MOG35-55	[79]
		GMM	NA	5–6 weeks	MBP Ac1-11	[80]
		C57BL/6 mice	F	7 weeks	MOG35-55	[81]
	Calcitonin (CT)	C57BL/6J mice	F	9–10 weeks	MOG35-55	[82]
		C57BL/6 mice	M & F	8–12 weeks	MOG35-55	[83]
		C57BL/6 mice	F	6–8 weeks	MOG35-55	[85]
		C57BL/6 mice	NA	6–10 weeks	MOG35-55	[87]
		C57BL/6, SJL, and GMM mice	F	7–8 weeks	MOG35-55	[88]
Parathyroid Glands	Parathyroid hormone (PTH)	GMM	M & F	5–6 weeks	MOG	[89]

Abbreviations: gpSCH – guinea pig Spinal Cord Homogenate; rSCH – rat Spinal Cord Homogenate; rrMOG – recombinant rat Myelin Oligodendrocyte Glycoprotein; DA – Dark Agouti rats; GMM – Genetically manipulated mice.

## 6 Parathyroid glands

### 6.1 Parathyroid hormone (PTH)

Given the fact that 1,25-dihydroxyvitamin D3 (1,25-(OH)_2_D3) regulates the calcium level in blood by increasing calcium absorption from the small intestine, releasing calcium from bone, and increasing the reabsorption of calcium in the kidney through a PTH-mediated mechanism, Meehan et al. examined the role of PTH in EAE development [89]. They used a 25-hydroxyvitamin D3-1alpha-hydroxylase knockout mouse which is responsible for the synthesis of 1,25-(OH)_2_D3. This strain of mice was then treated with PTH to induce hypercalcemia, without changing the circulating levels of 1,25-(OH)_2_D3. This PTH-mediated hypercalcemia inhibited EAE development in female, but not male mice. In contrast, once hypercalcemia was suppressed by diet, PTH administration no longer prevented EAE, suggesting that hypercalcemia prevented EAE after disease induction in female mice.

A summary of EAE animal models used to study parathyroid gland hormones is given in Table 3.

## 7 Bone

### 7.1 Osteocalcin (OCN)

It has been reported that the serum level of osteocalcin (OCN) has a negative correlation with the severity of EAE. Indeed, Ghareghani et al. reported that OCN serum level significantly decreased in EAE model, while medications which ameliorated EAE severity were associated with an increase in osteocalcin [90]. For instance, the serum level of osteocalcin significantly increased in EAE mice treated with cyclosporine A and 1α,25-(OH)_2_D3 (active form of Vitamin D) [91] or melatonin [90], but was insignificantly increased in 1,25(OH)_2_D3-treated EAE mice [92].

### 7.2 Lipocalin 2

Berard et al. used an Affymetrix gene array to track the alterations in the gene encoding Lipocalin 2, involved in matrix metalloproteinase (MMP) stabilization, glial activation, and cellular iron flux [93]. Lipocalin 2 gene expression increased in monocytes and reactive astrocytes by about 60-fold at the onset of EAE, while its receptor 24p3R was also shown to be expressed by monocytes, macrophages/microglia. Importantly, Lipocalin 2-deficent mice showed more severe EAE, in comparison to wild-type mice. In accordance, another study by Nam et al. demonstrated that EAE has been suppressed in Lipocalin 2^−/−^ mice [94]. In addition, Lipocalin 2-treated glial cells, isolated from Lipocalin 2^−/−^ EAE mice, had higher levels of proinflammatory cytokines, MMP-9, and chemokines. These findings highlighted that Lipocalin 2 is a critical mediator of autoimmune inflammation in EAE pathogenesis. Some pharmacological studies examined the effect of EAE medications on the expression of Lipocalin 2. For instance, increased levels of Lipocalin 2 were seen at active phases, onset and relapse, in blood-CSF sample as well as choroid plexus (CP) of EAE mice [95]; however, administration of Natalizumab, a well-known medication for MS [96], ameliorated the severity of EAE by modulating and normalizing CSF Lipocalin 2, while markedly reducing its astrocytic expression. Similarly, Ebrahimi-Kalan et al. showed the inhibitory effect of MS14, an herbal-marine drug with anti-inflammatory activity, on the severity of EAE through reduction of Lipocalin 2 [97].

### 7.3 Erythropoietin (EPO)

The neuroprotective role of erythropoietin in EAE was shown for the first time by Brines and colleagues who administrated the recombinant human erythropoietin (r-Hu-EPO), 3 days after immunization until day 18, causing a delay in EAE onset and reduction of peak clinical scores, in comparison to untreated-EAE rats [98]. It is well-known that astrocytes and microglia play a substantial role in MS progression, as activated and proliferating forms of these cells have been observed in demyelinated areas [99]. Agnello and colleagues reported that EPO considerably reduces both glial proliferation and activation and subsequently diminishes inflammation through reducing spinal cord TNF-α and IL-6 [100]. Importantly, Li and colleagues reported that erythropoietin also reduces the blood-brain barrier (BBB) leakage and major histocompatibility complex class II (MHC-II) in the spinal cord [101]. This reduced level of MHC-II was further observed by Zhang et al., who demonstrated that EPO suppresses the levels of IFN-γ and IL-17 both in peripheral splenic cells and CNS-infiltrating cells [102]. Similarly, it was shown that EPO improved neurological functional recovery in EAE mice by increasing the level of BDNF^+^ cells and proliferation of oligodendrocyte progenitor cells (OPCs), stimulating oligodendrogenesis, and by diminishing proinflammatory infiltration [103]. In line, combination therapy of methotrexate and EPO revealed a stimulating effect on oligodendrogenesis [104].

On the other hand, to clarify the best time of EPO administration in EAE, Savino and colleagues [105] showed that EPO therapy works in all stages of EAE; however, its efficiency is reduced once it is administrated late, 15 days after the onset of symptoms. The effects of EPO on hematocrit and EAE pathological outcomes were investigated by Yuan et al., using truncated small linear or cyclic EPO-derived peptides from various domains of the full-length EPO [106]. The 19-mer JM-4 peptide showed the best potential to treat EAE without producing hematocrit alterations in EAE mice, similar to the full-length EPO, and modulated immune/inflammatory reaction in EAE. EPO induces its neuroprotective role via signaling to several critical subsets of immune cells residing in the peripheral lymphatic system. Indeed, EPO reduced the proliferation of MOG-specific T cells *in vitro*, suppressed T cell cytokine production, reduced the total number of mononuclear and T cells in EAE peripheral lymph nodes, increased the expansion of peripheral Tregs, and inhibited the polarization of Th17 cells in EAE mice.

It has been shown that expression of the inhibitor of metalloproteases (TIMPs) is increased in astrocytes forming the BBB and surrounding demyelinated area [107], while TIMP-1-deficient mice have elevated levels of demyelination and microglial activation in EAE mice [108]. Thorne et al. showed that darbepoetin alfa therapy, an EPO analogue, reduced the clinical scores of EAE mice, accompanied with an increased number of TIMP-1-expressing astrocytes, both in the brain and spinal cord [109]. Although TIMP-1-deficient mice showed severe EAE outcomes, compared to wild-type, darbepoetin alfa therapy could not affect EAE severity in TIMP-1^−/−^ mice, highlighting TIMP-1 importance in EPO therapy of EAE. Similarly, ARA290, a nonerythropoietic analogue, has been shown to considerably ameliorate the severity of EAE [110]. In accordance, the expression of EPO was shown to be induced in spinal cord of EAE mice, clearly localized to motor neurons, and the authors suggested that EPO should be viewed as part of the inflammatory/anti-inflammatory network in MS [111]. On the other hand, EPO therapy in EAE mice induced endogenous heme oxygenase-1 (HO-1), an anti-oxidative stress protein, a possible factor in diminishing the inflammatory responses involved in EAE [112]. Recently, Moransard et al. used transgenic mouse strains to investigate whether EPO primarily applies its effects in the CNS or the periphery [113]. Although EAE onset did not differ between control and two transgenic EAE mice with overexpressed r-Hu-EPO either in CNS only (tg21) or systemically (tg6), their progression markedly increased in transgenic mice compared to controls. However, no peripheral immunomodulatory effects were seen in tg21 strain whose modulation was limited to a reduction in CNS macrophages. In contrast, macrophages were upregulated in the CNS, but not the periphery, of tg6 strain.

On the other hand, Sattler et al. showed on EAE mice that EPO promotes the survival and function of retinal ganglion cells (RGCs), the neurons that form the axons of the optic nerve, through three independent intracellular signaling pathways of p-Akt, p-MAPK 1 and 2, and Bcl-2 [114]. In accordance, the work by Diem and colleagues revealed that coadministration of EPO and high dose methylprednisolone, a steroid medication in MS, had relatively higher potential to induce neuroprotection in RGCs and optic nerves, compared to respective monotherapies [115].

Long-term effects of EPO were investigated, for the first time, by treating EAE mice using JM4, an EPO-derived small peptide, which is free from any side effects. Following 12 days of JM4 therapy, they observed a reduction in disease severity after day 60 until day 100 postimmunization [116].

### 7.4 FGF-23 (phosphatonin)

No studies were found concerning FGF-23 in EAE model.

A summary of EAE animal models used to study bone hormones is given in Table 4.

**Table 4:** A summary of EAE animal models used for hormones in the bone

Tissue	Hormone	Animal and strain of EAE model	Gender	Age	Immunogen for EAE induction	Reference
Bone	Osteocalcin (OCN)	C57BL/6 mice	F	6–8 weeks	MOG35-55	[90]
		SJL/ola mice	M	NA	MSC	[91]
		SJL mice	F	8–10 weeks	PLP139-151	[92]
	Lipocalin 2	GMM	F	8–11 weeks	MOG35-55	[93]
		GMM	NA	7–8 weeks	MOG35-55	[94]
		SJL mice	F	6–8 weeks	PLP139-151	[95]
		C57BL/6 mice	F	8–10 weeks	MOG35-55	[97]
	Erythropoietin (EPO)	LWS rat	F	6–8 weeks	MBP	[98]
		LWS rat	F	6–8 weeks	gpMBP	[100]
		C57BL/6 mice	F	8–14 weeks	MOG35-55	[101]
		C57BL/6 mice	F	8–10 weeks	MOG35-55	[102]
		SJL/J mice	F	8–10 weeks	PLP139-151	[103]
		C57BL/J6 mice	F	NA	MBP	[104]
		C57BL/6 mice	F	6–8 weeks	MOG35-55	[105]
		SJL & C57BL/6 mice	F	6–8 & 8–10 weeks	PLP139-151 & MOG35-55	[106]
		GMM	M & F	Adult	MOG35-55	[109]
		LWS rat	M	8–12 weeks	MBP68-84	[110]
		C57BL/6 mice	F	6–8 weeks	PLP139-151	[111]
		C57BL/6 mice	NA	6–8 weeks	MOG35-55	[112]
		GMM	M & F	8–12 weeks	MOG35-55	[113]
		BN rats	F	8–10 weeks	rrMOG	[114,115]
		SJL/J mice	F	8–10 weeks	PLP139-151 or MOG35-55	[116]

Abbreviations: gpMBP – guinea pig Myelin Basic Protein; MSC – Mouse Spinal Cord; rrMOG – recombinant rat Myelin Oligodendrocyte Glycoprotein; PLP – Proteolipid Protein; BN – Brown Norway; SJL/J – Swiss Jackson Laboratory/Jackson mice; GMM – Genetically manipulated mice.

## 8 The adrenal glands

### 8.1 Adrenal cortex

#### 8.1.1 Glucocorticoids (e.g., cortisol)

Several studies have shown that EAE is accompanied by a reduction in the intensity of cortisol metabolism under conditions of *in situ* perfusion of the liver by solutions containing cortisol in different concentrations as well as in lymphocytes from the cervical lymph nodes of guinea pigs [117,118,119].

#### 8.1.2 Mineralocorticoids (e.g., aldosterone)

Herrada and colleagues demonstrated that administration of deoxycorticosterone acetate (DOCA), a stable mineralocorticoid analogue of aldosterone, leads to an exacerbation of EAE severity through promotion of Th17 immunity. It is well-known that high levels of aldosterone lead to hypertension and cardiovascular disease where T cell immunity is involved [120].

### 8.2 Adrenal medulla

#### 8.2.1 Adrenaline (epinephrine)

Wesselmann and colleagues investigated splenic catecholamine in EAE and showed that elevated concentrations of epinephrine were not statistically significant, compared to controls [121]. Since epinephrine action is mediated by β adrenergic receptors, an antagonist of β adrenergic receptors called nadolol was used in EAE model and it was found that the suppression of epinephrine did not affect the clinical outcomes of EAE [122]. The function of epinephrine was recently further investigated by Yan and colleagues. They observed a high expression of the rate-limiting enzyme of epinephrine synthesis, phenylethanol N-methyltransferase (PNMT), in tissue-resident TH17 cells of EAE mice, not in other effector T cell subsets or regulatory T cells. They used a knockout mouse model for PNMT expressing immune cells. Although they observed a lower level of immune cell infiltration in these mice compared to normal EAE mice, the clinical scores did not show significant differences [123].

#### 8.2.2 Noradrenaline (norepinephrine)

White and colleagues showed that norepinephrine decreased sharply in the spinal cord of EAE rats, especially in the lumbar region [124]. In addition, norepinephrine depletion was more evident in lumbosacral and craniothoracal region of spinal cord EAE rats [125]. In contrast, development of acute EAE was suppressed by administration of neurotransmitter-depleting agents [126]. Indeed, depletion of norepinephrine using 6-hydroxydopamine suppressed EAE possibly through deletion of effector amplification mechanism and/or triggering a T suppressor cell mechanism [127]. However, another study suggested that this observation could be due to alteration in the blood-spinal cord barrier function and/or CNS blood flow [128]. In contrast, a study demonstrated that selective elevation of CNS NA levels could provide benefits in EAE, as increasing the norepinephrine levels using atomoxetine, a norepinephrine reuptake inhibitor, or l-threo-3,4-dihydroxyphenylserine (l-DOPS), a synthetic norepinephrine precursor, did not affect clinical scores and prevented further worsening of EAE, respectively [129]. In addition, Polak and colleagues suggested that raising norepinephrine levels is an efficient method to treat EAE and MS [130]. It is well-known that the primary source of norepinephrine are tyrosine hydroxylase-positive neurons in the Locus coeruleus, and that dysregulation of neurons was observed in EAE and MS. Therefore, Vindeburnol was selectively used to increase Locus coeruleus neuronal viability or activity which resulted in a reduction in astrocyte activation in the Locus coeruleus, hypertrophy of tyrosine hydroxylase-positive neurons, and elevation in spinal cord content of norepinephrine and genes involved in Locus coeruleus survival and maturation [131].

Moreover, Leonard et al. reported an inverse correlation between hypothalamic norepinephrine and corticosterone serum level, both at the peak clinical scores of EAE and in norepinephrine-depleted peripheral nervous system EAE mice [132]. However, a direct correlation was found between corticosterone serum levels and EAE severity, while the striatum showed no change in its content of norepinephrine in EAE mice [133]. Finally, Shaked and colleagues suggested that the transcription factor Nr4a1 regulates the production of norepinephrine in macrophages, as Nr4a1-deficient myeloid cells in mice led to an increase in norepinephrine and EAE severity [134]. In contrast, myeloid-specific deletion of tyrosine hydroxylase, the rate-limiting enzyme in norepinephrine production, demonstrated a protective role against EAE. Although males have a stronger sympathetic nervous system, Vujnović and colleagues reported that β-adrenoreceptor-mediated signaling plays a key role in sexual dimorphism in primary CD4^+^ T-cell responses in EAE rats. Indeed, rats treated with Propranolol, a nonselective β-adrenergic receptor antagonist, showed higher noradrenaline concentration in draining lymph node (dLN) in males, compared to females [135]. Furthermore, they did another study on α 1-adrenoceptor and suggested that this receptor is also involved in interactions between subtypes of CD4^+^ T cells and antigen-presenting cells [136]

A summary of EAE animal models used to study adrenal gland hormones is given in Table 5.

**Table 5:** A summary of EAE animal models used for hormones in the adrenal glands

Tissue	Source	Hormone	Animal and strain of EAE model	Gender	Age	Immunogen for EAE induction	Reference
The Adrenal Glands	Adrenal cortex	Glucocorticoids (e.g., cortisol)	NA	NA	NA	NA	[117,118,119]
		Mineralocorticoids (e.g., aldosterone)	GMM	F	6–8 weeks	MOG35-55	[120]
	Adrenal medulla	Adrenaline (Epinephrine)	LWS rat	M	3 months	gpMBP	[121]
			B10.PL mice	M	4–6 weeks	gpMBP	[122]
			Th-CKO mice	F	10–12 weeks	MOG35-55	[123]
		Noradrenaline (Norepinephrine)	LWS rat	M	NA	lrSCH	[124]
			LWS rat	F	NA	gpSCH	[125,132]
			LWS rat	F	NA	rSCH	[126]
			LWS rat	M	NA	gpMBP	[127]
			LWS rat	M	4 weeks	gpMBP	[128]
			C57BL/6 mice	F	6–8 weeks	MOG35-55	[129,130,131]
			C57B1 mice	M	8–12 weeks	MOG	[133]
			DA rat	M & F	3 months	rSCH	[135]
			DA rat	M & F	3 months	rSCH	[136]

Abbreviations: gpSCH – guinea pig Spinal Cord Homogenate; lrSCH – lewis rat Spinal Cord Homogenate; rSCH – rat Spinal Cord Homogenate; DA – Dark Agouti rats; GMM – Genetically manipulated mice.

## 9 Stomach, pancreas, and intestine

### 9.1 Cholecystokinin (CCK)

The therapeutic potential of GB-115 compound (*N*-phenylhexanoyl-glycyl-l-tryptophan amide), a dipeptide cholecystokinin receptor antagonist, was demonstrated by suppression of ROS production, improved spontaneous locomotor activity, and reduced edema and neutrophil infiltration of the perivascular space of the brain [137].

### 9.2 Neuropeptide Y

The role of neuropeptide-Y in EAE was first reported by Bedoui and colleagues [138]. Neuropeptide-Y decreases the susceptibly of mice to EAE induction and was shown to exert its pleiotropic functions via the activation of several G-protein coupled receptor subtypes such as Y(1) receptor, but not Y(5). Moreover, EAE induction was facilitated by administration of Y(1) receptor antagonist. In contrast, the immunomodulatory action of neuropeptide-Y in DA EAE rats showed that CD28 and CD80/CD86 costimulatory molecules acted as a target for neuropeptide-Y-mediated amelioration in EAE through Y(2) and Y(5) receptors [139]. Moreover, Brod and Bauer showed that ingested (oral) neuropeptide-Y inhibits CNS inflammation in EAE mice by diminishing Th17 and Th1-like cytokines and elevating Th2-like cytokines within the CNS [140].

### 9.3 Ghrelin

Theil et al. examined the role of ghrelin in EAE pathogenesis and showed that although its administration ameliorates the severity of EAE clinical scores, it had no beneficial effect in reducing the numbers of inflammatory cells in the spinal cord [141]. Indeed, this has been determined by counting the number of NK cells (NK1.1^+^CD3^−^), B cells (CD19^+^), NKT cells (NK1.1^+^CD3^+^), macrophages (F4/80^+^), and CD25^+^ FOXP3^+^ cells (in the CD4^+^ T cell population) in the spinal cord lesions. Theil et al. suggested that monocytes could be potential targets in the ghrelin-mediated EAE inhibition. While the levels of TNF-α, IL-1β, and IL-6 significantly decreased in the spinal cord of ghrelin-treated mice, only TNF-α mRNA levels decreased in their spleens, whereas no significant changes were seen in any of the cytokines in the lymph node or thymus of ghrelin-treated mice. To investigate which cells were important in the ghrelin-mediated suppression of EAE, mRNA levels of IL-1β, IL-6, and TNF-α were assessed in macrophages, microglia, and T cells; however, they only showed marked changes in microglia, suggesting that it might play a key role in ghrelin-mediated suppression of EAE. The effect of ghrelin was further investigated by Liu and colleagues who observed a reduction in the levels of inflammatory cytokines and factors involved in NLRP3 inflammasome signaling and Pyroptosis [142].

### 9.4 Serotonin

An early study demonstrated that a segment of MBP is composed of one of the 5-hydroxytryptamine (5-HT; serotonin) receptors in the CNS which played an important role in EAE [143]. Later, it was reported that CNS 5-HT neurotransmission is impaired in EAE rats with hind limb paralysis [144] which was correlated with motor deficits manifested during the acute paralytic stage of EAE [145]. In line, administration of some drugs led to modifications in endogenous serotonin levels within the body. Treatment of EAE guinea pigs with serotonin suppressor, especially the antihypertensive medication reserpine, led to quick death of EAE animals. However, serotonin enhancer medication, particularly imipramine hydrochloride antidepressant, showed better survival, even longer than controls [146]. Moreover, using serotonin antagonist and inactivation of neurogenic 5-HT receptors significantly reduced the severity of EAE [147,148,149,150]. In contrast, the serotonin antagonist Methysergide did not inhibit the severity of EAE in rabbits when administrated 6 days postimmunization [151].

On the other hand, it was reported that serotonin axons were swollen and distorted at early stages of EAE and get worse when neurological scores increase. Interestingly, the severity of axonal damage correlated with the severity of inflammation and the injured axons were located adjacent to blood vessels or the pial surface, sites at which inflammation occurs during EAE [39]. Moreover, reduced levels of serotonin and the serotonin metabolite, 5-hydroxyindoleacetic acid, were shown in EAE rats which suggested that the reduction is probably a consequence of the damage to descending 5-HT axons [152]. Another study reported that factors associated with spinal cord inflammation may be responsible for the bulbospinal monoaminergic axonal damage in EAE. Indeed, prazosin therapy, an alpha 1-adrenergic antagonist, reduced EAE severity and pathological outcomes [153].

Preventive and therapeutic outcomes were found for antidepressant drugs on EAE such as the use of selective serotonin/norepinephrine reuptake inhibitors (SNRI) sertraline [154], fluoxetine [155,156], and fluvoxamine [157]. Also, immunomodulatory effects of antipsychotic agents were reported in EAE such as the use of risperidone, known to antagonize serotonin 5-HT2A receptors [158], and clozapine [159]. Moreover, monoamine oxidase inhibitor phenelzine therapy, an antidepressant and anxiolytic drug, was reported as an efficient strategy to ameliorate EAE severity [160,161]. Furthermore, attenuation of EAE was shown in mice lacking the 5-HT transporter (5-HTT), which was more prominent in females [162]. Finally, elevated levels of serotonin were found in the brain striatum of EAE mice [133].

### 9.5 Amylin, secretin, fibroblast growth factor 19, PYY3-36, and incretins

No studies were found concerning the above hormones in EAE model.

A summary of EAE animal models used to study hormones of the stomach, pancreas, and intestine is given in Table 6.

**Table 6:** A summary of EAE animal models used for hormones in stomach, pancreas, and intestine

Tissue	Hormone	Animal and strain of EAE model	Gender	Age	Immunogen for EAE induction	Reference
Stomach, Pancreas, and Intestine	Cholecystokinin (CCK)	C57Bl/6 mice	F	5–6 weeks	MOG35-55	[137]
	Neuropeptide Y	B6 & SJL/J mice	F	6–10 weeks	MOG35-55 & PLP139151	[138]
		DA rat	M	7 months	gpSCH	[139]
		C57BL/6	F	6–8 weeks	MOG35-55	[140]
	Ghrelin	B6 mice	F	6–10 weeks	MOG35-55	[141]
		CD rat	F	NA	gpSCH	[142]
	Serotonin	LWS rat	M	NA	lrSCH	[39,152,153]
		Guinea pigs	NA	Young adult	bBH	[143]
		CRW-L rats	M	NA	w-lrSCH	[144]
		guinea pigs	NA	NA	gpMBP	[146,148]
		LWS rat	F	NA	gpBH	[149]
		LWS rat	M	NA	gpMBP	[150]
		White rabbits	F	Adult	rSCH	[151]
		C57/bl mice	F	NA	MOG	[154]
		WSR rats	F	6–8 weeks	gpSCH	[155]
		SJL/J mice	F	8–10 weeks	MBP AC1-11 & PLP139-151	[156]
		LWS rat	F	8–12 weeks	gpSCH	[157]
		C57BL/6 mice	F	8–12 weeks	MOG35-55	[158,159]
		C57BL mice	F	NA	MOG35-55	[160,161]
		C57BL/6 mice	M & F	2–5 months	MBP & MOG	[162]
		C57B1 mice	M	8–12 weeks	MOG	[133]

Abbreviations: gpSCH – guinea pig Spinal Cord Homogenate; gpBH – guinea pig Brain Homogenate; lrSCH – lewis rat Spinal Cord Homogenate; w-lrSCH – wistar-lewis rats Spinal Cord Homogenate; bBH – bovine Brain Homogenate; rSCH – rat Spinal Cord Homogenate; PLP – Proteolipid Protein; DA – Dark Agouti rats; CRW-L – Charles River Wistar-Lewis rats; WSR – wistar rat; SJL/J – Swiss Jackson Laboratory/Jackson mice.

## 10 Liver

### 10.1 Insulin-like growth factor-1 (IGF-1)

Liu and colleagues showed that the levels of IGF-1 and GFAP, an astrocytic marker, are elevated in demyelination areas in EAE model [163]. These astrocytes co-expressed IGF-I with IGFBP-2, a member of the six IGF binding proteins that modulate IGF-1 actions, suggesting that astrocytic expression of IGF-1-related peptides could reduce the immune-mediated demyelination. Indeed, another study reported that IGF-1-induced reductions in immune cell responses can occur even in the absence of demyelination [164]. Similar studies further reported the beneficial role of IGF-1 in EAE and suggested that IGF-1 therapeutic activity is mediated by reducing EAE-induced blood-spinal cord barrier changes, modifying oligodendrocytes to increase myelin regeneration [165,166,167]. Using *in vivo* three-dimensional MR microscopy imaging technique, IGF-I was shown to reflect changes in stabilization or permeability of BBB and/or cell membranes to ameliorate EAE [168]. On the other hand, it has been suggested that coadministration of IGF-1 and IGFBP3 can enhance the efficiency of IGF-1 therapy in EAE [169].

In contrast, Cannella and colleagues were doubtful of IGF-1 being a good therapeutic agent in MS as they showed that administration of IGF-1 at different time points during the acute and chronic phases of EAE had different outcomes and even failed to enhance CNS myelin repair in EAE [170]. Similarly, Bilbao et al. showed that not only administration of recombinant human IGF-1 (rhIGF-1) stimulates regulatory T cells in culture, but it also ameliorates the severity of EAE. Indeed, they indicated that existence and function of IGF-1 receptor are the necessary factors to induce IGF-1 stimulatory effect on regulatory T cell proliferation [171]. In accordance, Genoud and colleagues showed that adeno-associated virus (AAV)-mediated delivery of IGF-1 in the spinal cord had no effect on EAE and even worsened the disease when injected after EAE induction [172]. Considering the fact that Th17 and Treg cells balance is disrupted in MS, which play a critical role in immune tolerance, DiToro and colleagues showed that increased aerobic glycolysis following activation of IGF1R leads to a higher Th17 cell differentiation than Treg cells, resulting in an increase in proinflammatory cytokines [173].

### 10.2 Thrombopoietin (TPO)

Although TPO, a hematopoietic growth factor, induced an increase in the number of white blood cells in TPO-treated EAE mice, the clinical scores of EAE severity did not differ either after immunization or after the disease reached its peak [174]. The mechanism of TPO’s effect on the immune response of EAE remains unknown.

### 10.3 Hepcidin

Iron accumulates in the CNS of MS patients and in EAE models [175,176]. Iron efflux from cells is mediated by the multi-pass membrane transporter ferroportin (Fpn) which is regulated posttranscriptionally by hepcidin, thus reducing iron efflux and leading to iron accumulation. Zarruk et al. showed that iron and ferritin elevation in the CNS of EAE mice was coincident with about 5-fold hepcidin mRNA increase at all stages of chronic and at the peak of relapsing-remitting EAE [177]. In addition, hepcidin was found in infiltrating macrophages as early as the onset stage in chronic EAE, suggesting that hepcidin-mediated internalization of the Fpn and disruption of iron efflux could be the cause of iron accumulation in macrophages/microglia. In accordance, Ćurko-Cofek et al. revealed that chronic iron accumulation affects the clinical course of DA EAE model and accelerated the onset of EAE; however, it accelerated the progression and severity of EAE in male rats [178]. This difference could be due to the variations in the expression of stress response proteins hepcidin and metallothioneins in female and male iron overloaded rats.

### 10.4 Angiotensinogen and betatrophin

No studies were found concerning these hormones in EAE model.

A summary of EAE animal models used to study liver hormones is given in Table 7.

**Table 7:** A summary of EAE animal models used for hormones in the liver and fat cells (adipocytes)

Tissue	Hormone	Animal and strain of EAE model	Gender	Age	Immunogen for EAE induction	Reference
Liver	Insulin-like growth factor-1 (IGF-1)	LWS rat	M	NA	gpSCH	[163,165,166,167]
		(PL * SJL)F1 mice	NA	8–10 weeks	gpMBP	[169]
		SJL/J mice	F	5–12 weeks	MBP	[170]
		C57BL/6 mice	NA	NA	MOG35-55	[172]
		C57BL/6 mice	F	5–12 weeks	MOG35-55	[173]
	Thrombopoietin (TPO)	SJL/J mice	F	5–6 weeks	PLP139-151	[174]
	Hepcidin	C57BL/6 mice	F	8–10 weeks	MOG35-55	[177]
		DA rats	M & F	7–8 weeks	bBH	[178]
Fat cells (adipocytes)	Leptin	GMM	F	8–10 weeks	MOG35-55	[179]
		SJL/J mice	M & F	4–6 weeks	PLP139-151	[180]
		SJL/J mice	F	6–8 weeks	PLP139-151	[181]
		GMM	F	8–12 weeks	MOG35-55	[183]
		SJL/J mice	F	6 weeks	PLP139-151	[184]
		GMM	F	8–10 weeks	MOG79-96	[185]
		GMM	F	8–10 weeks	MOG35-55	[186]
		SJL/J & C57BL/6J mice	F	10 weeks	PLP139-151 & MOG35-55	[187]
		C57BL/6J mice	NA	8–10 weeks	MOG	[188]
	Adiponectin	GMM	NA	NA	MOG35-55	[189]
		GMM	F	6–8 weeks	MOG35-55	[190]

Abbreviations: gpSCH – guinea pig Spinal Cord Homogenate; bBH – bovine Brain Homogenate; PLP – Proteolipid Protein; DA – Dark Agouti rats; SJL/J – Swiss Jackson Laboratory/Jackson mice; GMM – Genetically manipulated mice.

## 11 Fat cells (adipocytes)

### 11.1 Leptin

Matarese et al. used female C57BL/6J wild-type and leptin-deficient (ob/ob) mice to study the influence of leptin on EAE, with or without leptin replacement [179]. They showed that leptin plays a key role in the induction and development of EAE, as leptin-deficient mice were resistant to EAE induction; however, administration of recombinant leptin caused susceptibility to EAE induction and an increase in IFN-γ levels, accompanied by modifying Th1 proinflammatory immune responses and consequent reversal of Ig subclass production. Moreover, these observations were confirmed in a different type of EAE model using SJL (H-2s) female mice in which leptin treatment, before or after EAE onset, significantly exacerbated the disease [180]. In accordance, it was further demonstrated that blocking leptin with antibodies or with a soluble mouse leptin receptor chimera ameliorates the severity of EAE symptoms [181].

On the other hand, the effects of leptin on the kinetics of two models of chronic-progressive and relapsing-remitting EAE, using C57BL/6J (H-2^b^) and SJL/J (H-2^s^) mice strains, respectively, were investigated by Sanna et al. Results suggested that leptin is required for the induction and development of proinflammatory response in EAE. Hence, the authors introduced the modulation of leptin-mediated inflammation as an innovative therapeutic achievement particularly in the treatment of Th1 autoimmune diseases including MS. Moreover, histamine receptors type 3 (H3R) is involved in leptin action since H3R-deficient mice developed an obese phenotype related to increased levels of leptin [182]. Therefore, Musio et al. used HDC-deficient mice, which are unable to synthesize histamine, and showed that leptin levels were elevated, suggesting that histamine receptors are involved in regulating autoimmunity in the EAE, in which leptin plays a key role [183].

As shown above, conflicting studies exist about the role of leptin in EAE, being detrimental or beneficial. Recently, Wu et al. demonstrated that EAE is associated with a sharp increase in leptin receptor (ObR) in the reactive astrocytes of hippocampus and hypothalamus [184]. Astrocyte-specific GFAP mRNA and protein were both increased; however, ObRa mRNA was elevated only after resolution of EAE symptoms, whereas ObRb mRNA was decreased at the peak of EAE. Indeed, regulation of LepR mRNA in the hippocampus is subtype-specific with a late increase of LepRa that lags behind the increase of protein expression and a transient reduction of LepRb during the peak of the disease. In contrast, Mishra et al. showed that astrocytic leptin signaling plays a beneficial role in reducing disease severity [185]. Indeed, astrocyte-specific leptin receptor knockout mice had severe EAE symptoms, compared to wild-type, suggesting that astrocytic leptin signaling serves as a beneficial factor in clearing the infiltrating leukocytes and decreasing autoimmune destruction of the CNS. In accordance, endothelial leptin signaling in EAE showed that removal of leptin signaling, using endothelial leptin receptor-specific knockout mice, reduced EAE severity which emphasized that endothelial leptin signaling increases BBB dysfunction to exacerbate EAE [186].

Calorie-restriction or fasting improved EAE severity, which was concomitant with lower levels of leptin [187]. In order to study the role of energy in EAE development, hypoleptinemia was induced by keeping the mice in a fasting situation, with or without leptin injections for 48 h at the onset of EAE [188]. Fasted mice showed lower EAE clinical scores, compared to control-fed mice. Moreover, fasted mice receiving leptin injection during this period had more severe EAE symptoms than fasted mice without leptin injection. These observations demonstrated that leptin alone can reverse the protection against autoimmunity seen during fasting.

### 11.2 Adiponectin

Piccio et al. reported for the first time that adiponectin confers a neuroprotective role in EAE [189]. Indeed, adiponectin-deficient mice were used to induce EAE and showed that lack of adiponectin exacerbates EAE severity by inducing higher CNS inflammation, demyelination, and axonal injury. In fact, lack of adiponectin directly affects immune responses, by promoting T cell responses to antigen while Th1 profile was prominent. Moreover, adiponectin receptors 1 and 2 were found to be markedly expressed by murine lymph node cells and splenocytes. In the absence of adiponectin and in response to anti-CD3 stimulation, increased proliferation of ADPKO CD4^+^ T cell was obtained. Complementary to this study, Zhang et al. revealed the role and mechanism of adiponectin on pathogenic Th17 cells [190]. They reported that adiponectin-deficient mice enhance both Th1 and Th17 cell cytokines in the peripheral immune system and CNS of EAE mice as well as upregulated PPARγ and sirtuin 1 (SIRT1) and suppressed retinoid-related orphan receptor-γt (RORγt), a key transcription factor during the differentiation of Th17 cells. These results suggested that adiponectin modulates the immune response in EAE by inhibiting Th17 cell-mediated autoimmune CNS inflammation.

### 11.3 Betatrophin, retinol binding protein 4, and asprosin

No studies were found concerning these hormones in EAE model.

A summary of EAE animal models used to study hormones of the fat cells (adipocytes) is given in Table 7.

## 12 Conclusion

Based on investigations carried out on the endocrine system and EAE, most hormones show conflicting roles in susceptibility to EAE and on therapeutic targets for EAE. This could be due, among others, to different factors such as sampling in different phases of EAE progression, sex, age, strain of mice/rat models used, the type of EAE induction, and the concentration of hormones in therapeutic studies. While some studies reported the beneficial role of melatonin in EAE amelioration, others showed opposite and conflicting results and reported age- and dose-dependent response of EAE models to melatonin therapy. Indeed, it is suggested that the timing of melatonin administration leads to different outcomes in EAE. We strongly believe that studies on the endocrine system in EAE are still at their beginning and we are far away from understanding the role of hormones in EAE susceptibility; hence, further studies are required. New studies should consider the above-mentioned factors to achieve more precise results. For example, hormones or their receptor’s agonists/antagonists should be administered at different times of day/night, at different physiological and pharmacological doses, use mice or rats at different ages, and study the effects at different stages of EAE. This also can be considered for the studies investigating the effect of EAE induction on endocrine system function.
